# Limited Moderating Effect of Podcast Listening on Work Stress and Emotional Exhaustion Among Nurses During the COVID-19 Pandemic: Cross-Sectional Study

**DOI:** 10.2196/70640

**Published:** 2025-04-16

**Authors:** Lu Li Jung, Pei Chin Chou, Yu-Hua Wu

**Affiliations:** 1 Healthcare Information Management Institute of Healthcare Information Management, College of Management National Chung Cheng University Minxiong Township Taiwan; 2 Department of Respiratory Care Chang Gung University of Science and Technology Taipei Taiwan

**Keywords:** work stress, emotional exhaustion, podcasts, nurses, COVID-19, mental health

## Abstract

**Background:**

The COVID-19 pandemic placed unprecedented pressure on health care systems worldwide, significantly impacting frontline health care workers, especially nurses. These professionals faced considerable psychological stress from caring for patients with COVID-19 and the fear of spreading the virus to their families. Studies report that more than 60% (132/220) of nurses experience anxiety, depression, and emotional exhaustion, which adversely affect their mental health and the quality of care they provide.

**Objective:**

This study aimed to investigate the relationship between work-related stress and emotional exhaustion among nurses and to assess whether listening to podcasts moderates this association.

**Methods:**

A cross-sectional online survey was conducted between March 1, 2023, and March 31, 2023. A total of 271 clinical nurses, aged 20 years to 65 years, were recruited for the study. Participants were divided into 2 groups: experimental group consisting of regular podcast listeners (n=173) and control group comprising nonlisteners (n=98). Ethical approval for this study was obtained from the local ethics committee (IRB number YGHIRB20230421B). Validated scales were used to measure work stress, emotional dissonance, and emotional exhaustion. Data analysis included descriptive statistics, independent *t* tests, and structural equation modeling to examine the relationships between variables.

**Results:**

No statistically significant differences were found between the experimental and control groups in terms of overall work stress (mean difference=–0.09, 95% CI –0.31 to 0.13; *P=*.42) or emotional exhaustion (mean difference=0.07, 95% CI –0.15 to 0.29; *P=*.53). Emotional dissonance emerged as a significant predictor of emotional exhaustion in both the experimental (β=0.476, *P*<.001) and control (β=0.321, *P=*.01) groups. Nurses reporting higher workloads had significantly higher emotional exhaustion levels (experimental group: β=0.302, *P*<.001; control group: β=0.327, *P*=.002). Podcast listening demonstrated only a slight, nonsignificant moderating effect.

**Conclusions:**

Although podcasts alone may not significantly reduce work stress or emotional exhaustion among nurses, there was a potential, albeit limited, moderating effect of podcasts on emotional well-being. They could serve as a supplementary tool for emotional support. However, broader and more comprehensive interventions are required to address the underlying causes of stress and emotional exhaustion in this population. More in-depth exploration and recommendations are possible by analyzing the content and patterns of listening. Further research is needed to examine the long-term benefits of integrating podcasts with other digital tools for holistic stress management in health care settings.

## Introduction

### Background and Significance

Following the initial outbreak in December 2019, the COVID-19 pandemic rapidly spread across the globe, resulting in a severe public health crisis. The pandemic imposed immense pressure on health care systems worldwide, particularly affecting frontline health care workers who bear significant responsibilities and endure substantial psychological stress. These health care workers made great sacrifices and contributions to combat the pandemic, yet they faced challenges such as nursing staff shortages, which had a profound impact on the global health care sector. Nurses were not only tasked with caring for patients with COVID-19 but also worried about transmitting the virus to their families, further exacerbating their psychological stress and adversely affecting their mental health [1].

### Work Stress and Emotional Exhaustion Among Nurses

Studies indicate that more than 60% (132/220) of nurses experience anxiety, depression, and emotional exhaustion due to prolonged patient care, impacting both their physical and mental well-being, as well as the quality of patient care they provide [2]. For instance, research by Lai et al [3] revealed that 126 of 250 nurses (50.4%) exhibit symptoms of depression, 112 of 251 nurses (44.6%) experience anxiety, 85 of 250 nurses (34%) experience insomnia, and as many as 179 of 250 nurses (71.5%) report distress. These data highlight the issue of emotional exhaustion among nurses in high-pressure environments.

### The Potential Role of Digital Interventions in Stress Management

The mental health challenges faced by nurses have drawn attention to the need for strategies to improve their psychological well-being. Previous research has predominantly focused on improving work environments and workplace ethics, while few explored the efficacy of digital media interventions for managing stress within health care settings. These interventions range from mobile apps to web-based programs targeting diverse populations including adolescents, health care workers, and patients with cancer [4-7]. With the advancement of information and network technologies, new media tools such as podcasts have gained attention. Podcasts, as an unofficial platform that crosses institutional and organizational boundaries, offer anonymity and flexibility, making them a potential resource for helping nurses manage stress and emotions.

### The Rise of Podcasts and Their Use in Health Care

With the progression of information and network technologies, podcasts have emerged as a popular form of media in Taiwan. In our survey, 201 of 250 participants (80.3%) reported using the internet to access online news or lifestyle information. Podcasts, although introduced in the early 2000s, gained substantial popularity during the COVID-19 pandemic and became part of the stay-at-home economy [8]. Due to a feeling of companionship and flexibility, podcasts have become a popular companion media format. A report by Commonwealth Magazine [9] indicated that most podcast listeners use podcasts for relaxation and entertainment, while programs like “Med persona” cater to the personal needs of health care professionals and inspire their professional development.

### Research Gap and Study Rationale

Podcasts are becoming increasingly important for supporting the emotional health and resilience of nurses, especially in the face of the challenges posed by the COVID-19 pandemic. These podcast programs provide essential resources for health care professionals, offering guidance and shared experiences to help nurses cope with the emotional labor and psychological stress often encountered in their work. However, despite their growing popularity, there is limited empirical research investigating the actual effectiveness of podcasts in reducing work-related stress and emotional exhaustion among nurses. This study aimed to fill this research gap by systematically examining whether podcast listening can serve as a useful tool for emotional support and stress relief in nursing practice.

### Objectives and Research Questions

The primary objective of this study was to investigate the impact of work-related stress on emotional exhaustion among nurses and to determine whether listening to podcasts can mitigate this effect. The study aimed to provide empirical evidence regarding the effectiveness of podcasts as a tool for emotional management and stress relief among nursing staff.

To achieve this objective, we focused on 2 main research questions: (1) Can listening to podcasts help nurses manage their emotions and alleviate work -related stress? and (2) Can podcasts modulate the level of emotional exhaustion among nurses?

By addressing these questions, the study aimed to gain a deeper understanding of the potential benefits of incorporating podcasts into stress management and emotional support programs for nurses, ultimately enhancing their overall well-being and job performance.

## Methods

### Study Design and Participants

The study used convenience sampling to ensure the accuracy and reliability of the study results. The participants were clinical care providers aged between 20 years and 65 years. The participants were divided into 2 groups: experimental group consisting of nurses who regularly listened to podcasts and control group comprising nurses who did not listen to podcasts. This grouping facilitated a comparative analysis of the potential impact of podcast listening on alleviating work-related stress and emotional exhaustion among nurses.

Exclusion criteria included any hearing impairments, ensuring that the selected sample accurately met the study’s requirements.

### Data Collection Procedures

During the recruitment process, participants in the experimental group were introduced to the study via a recorded message played by podcast hosts. This message explained the study's purpose, objectives, and instructions for completing the questionnaire. Participants in the control group received the same information in written form on the introduction page of the online survey platform. Participants were assured that their data would be used solely for research purposes, their privacy would be protected, and their participation was voluntary. They were informed their rights would not be affected if they chose not to participate. Those who agreed to participate were asked to sign a consent form and were then provided with a link to the online survey.

Data collection was conducted through a noninvasive online questionnaire. Participants were informed that they could withdraw from the study at any time if they felt uncomfortable. The questionnaire was administered by experienced researchers, and all responses were securely stored in a cloud database. Participants’ identities were anonymized using research codes to protect their privacy, in compliance with personal data protection laws and relevant regulations.

If participants felt any discomfort while completing the questionnaire, they were allowed to terminate their participation at any time.

### Statistical Analysis

During the study period, from March 1, 2023, to March 31, 2023, a total of 271 valid questionnaires were collected. The experimental group consisted of 173 nurses who regularly listened to podcasts, and the control group consisted of 98 nurses who did not listen to podcasts. The analysis focused on demographic attributes such as age, marital status, number of children, educational background, nursing experience, department, position, level of advancement, emotional disposition, and podcast listening habits.

This descriptive analysis provides a comprehensive overview of the demographic and occupational characteristics of the study participants, laying the foundation for further analysis of the relationships between these variables and the outcomes of interest.

### Ethical Considerations

All procedures of this study adhered to ethical guidelines. Contact information for the primary researcher and detailed instructions for submitting responses were provided. Informed consent was obtained from all participants, and all data were anonymized. No incentives nor rewards were offered. Ethical approval for this study was granted by the Yuan's General Hospital Ethics Committee (Institutional Review Board approval number: YGHIRB20230421B).

## Results

### Participant Characteristics

A total of 271 nurses participated in the study. Most participants were aged between 21 years and 39 years (187/271, 69%) and held a university-level education (221/271, 81.5%). The majority were unmarried (144/271, 53.1%), had no children (159/271, 58.7%), and had between 2 years and 20 years of nursing experience (205/271, 75.7%). Most held clinical nursing positions (229/271, 85.4%) and were distributed across various hospital departments, including the emergency department (22/271, 8.1%) and internal medicine (31/271, 11.4%) and surgical (54/271, 19.9%) wards. In terms of emotional disposition, most participants reported having a positive outlook (212/271, 78.2%). Among podcast listeners, the majority had been listening for less than 3 months (80/173, 46.1%). Detailed demographic information is presented in Table 1.

**Table 1:** Demographic data of the study participants (n=271).

Variable			Results, n (%)
**Age (years)**			
	21-29	90 (33.2)	
	30-39	97 (35.8)	
	40-49	65 (24)	
	50-59	18 (6.6)	
	60-69	1 (0.4)	
**Marital status**			
	Unmarried	144 (53.1)	
	Married	121 (44.6)	
	Divorce	6 (2.2)	
	Widowed	0 (0)	
	Separated	0 (0)	
**Education**			
	Specialist	20 (7.4)	
	2-year junior college program	16 (5.9)	
	University	221 (81.5)	
	Master	13 (4.8)	
	PhD	1 (0.4)	
**Nursing experience (years)**			
	0-1	22 (8.1)	
	2-10	123 (45.4)	
	11-20	82 (30.3)	
	21-30	34 (12.5)	
	31-40	9 (3.3)	
	41-50	1 (0.4)	
**Number of children**			
	0	159 (58.7)	
	1	41 (15.1)	
	2	62 (22.9)	
	3	9 (3.3)	
	>4	0 (0)	
**Service department**			
	Outpatient department	47 (17.3)	
	Emergency Department	22 (8.1)	
	Operating room	19 (7)	
	Intensive Care Unit	39 (14.4)	
	Medical Ward	31 (11.4)	
	Surgical Ward	54 (19.9)	
	Obstetrics and gynecology	18 (6.6)	
	Pediatrics	1 (0.4)	
	Other single	40 (14.8)	
**Emotional personality**			
	Positive emotions	212 (78.2)	
	Negative emotions	59 (21.8)	
**Nursing duties**			
	Nurse	229 (84.5)	
	Nursing team leader	18 (6.6)	
	Deputy director of nursing	6 (2.2)	
	Head nurse	17 (6.3)	
	Nursing supervision	1 (0.4)	
	Nursing director	0 (0)	
**Advanced level**			
	New staff	2 (0.7)	
	<1 year of clinical work experience	25 (9.2)	
	>1 year of clinical work experience, completed the first-year clinical competency training for nursing staff, and passed the review successfully	41 (15.1)	
	>2 years of clinical work experience, completed the second-year clinical competency training for nursing staff, and passed the review successfully	142 (52.4)	
	>3 years of clinical work experience, completed the third-year clinical competency training for nursing staff, and passed the nursing association's case report review successfully	38 (14)	
	>4 years of clinical work experience, completed the fourth-year clinical competency training for nursing staff, and passed the nursing association's administrative project review successfully	23 (8.5)	
**Podcast listening habits** **(n=** **173** **)**			
	1 month	46 (26.6)	
	3 months	34 (19.5)	
	6 months	18 (10.4)	
	1 year	24 (13.9)	
	1-3 years	32 (18.5)	
	3-5 years	13 (7.5)	
	5-10 years	5 (2.8)	
	>10 years	1 (0.6)	

Age (χ^2^_4_=49.158, *P=*.09; Table 2) and marital status (χ^2^_2_=2.223, *P*=.33) were not significantly different between the experimental and control groups. The number of children was significantly different between the 2 groups (χ²_2_=12.215, *P*=.007). In the experimental group (ie, nurses who regularly listen to podcasts), a higher proportion of participants were childless (61/98, 62%), whereas in the control group, a greater number of nurses had children (75/173, 43.4%). This difference may impact how nurses cope with work stress and experience emotional exhaustion, as studies have shown that nurses with children face additional family caregiving responsibilities, which may exacerbate their psychological stress. Additionally, since the number of children was a significant factor, it should be considered in the interpretation of the results to avoid potential confounding effects. There were no significant differences between the groups in educational level (χ^2^_3_=5.742, *P*=.22), nursing experience (χ^2^_4_=36.885, *P*=.38), department (χ^2^_5_=14.571, *P*=.07), position (χ^2^_2_=1.911, *P*=.75), advancement level (χ^2^_2_=2.058, *P=*.84), and emotional disposition (χ^2^_1_=0.512, *P*=.47). Independent *t* tests revealed no significant differences between the experimental and control groups regarding workload (*t*_269_=–.879, *P*=.51) and emotional exhaustion (*t*_269_=0.295, *P*=.30). These findings suggest that podcast listening did not significantly affect the levels of work stress or emotional exhaustion among the nurses in this study (Tables 2 and 3).

**Table 2:** Comparison of the distribution of individual variables between the 2 groups.

Variables			Experimental group (n=98), n (%)		Control group (n=173), n (%)		χ^2^ (*df*)			*P* value	
**Age (years)**								49.158 (4)			.09
	21-29	41 (41.9)		49 (28.3)							
	30-39	36 (36.7)		61 (35.3)							
	40-49	16 (16.3)		49 (28.3)							
	50-59	5 (5.1)		13 (7.5)							
	60-69	0 (0)		1 (0.6)							
**Marital status**								2.223 (2)			.33
	Unmarried	57 (58.2)		87 (50.3)							
	Married	40 (40.8)		81 (46.8)							
	Divorced	1 (1)		5 (2.9)							
	Widowed	0 (0)		0 (0)							
	Separated	0 (0)		0 (0)							
**Number of children**								12.215 (3)			.007
	0	61 (62.2)		98 (56.6)							
	1	21 (21.4)		20 (11.6)							
	2	12 (12.2)		50 (28.9)							
	3	4 (4.1)		5 (2.9)							
	>4	0 (0)		0 (0)							
**Education**								5.742 (4)			.22
	specialist	8 (8.2)		12 (6.9)							
	2-year junior college program	7 (7.1)		9 (5.2)							
	University	82 (83.7)		139 (80.3)							
	Master	1 (1)		12 (6.9)							
	PhD	0 (0)		1 (0.6)							
**Nursing experience (years)**								36.885 (5)			.38
	0-1	12 (12.2)		10 (5.8)							
	2-10	45 (46)		78 (45.1)							
	11-20	32 (32.7)		50 (29)							
	21-30	7 (7.1)		27 (15.6)							
	31-40	2 (2)		7 (4)							
	41-50	0 (0)		1 (0.5)							
**Service department**								14.571 (7)			.07
	Emergency department	18 (18.4)		29 (16.8)							
	Operating room	2 (2)		20 (11.6)							
	Intensive care unit	8 (8.2)		11 (6.4)							
	Medical ward	13 (13.3)		26 (15)							
	Surgical ward	13 (13.3)		18 (10.4)							
	Obstetrics and gynecology	25 (25.5)		29 (16.8)							
	Pediatrics	9 (9.2)		9 (5.2)							
	Other single	0 (0)		1 (0.6)							
**Nursing duties**								1.911 (3)			.75
	Nurse	85 (86.7)		144 (83.2)							
	Nursing team leader	7 (7.1)		11 (6.4)							
	Deputy director of nursing	2 (2)		4 (2.3)							
	Head nurse	4 (4.1)		13 (7.5)							
	Nursing supervision	0 (0)		1 (0.6)							
	Nursing director	0 (0)		0 (0)							
**Advanced level**								2.058 (5)			.84
	New staff	1 (1)		1 (1)							
	<1 year of clinical work experience	11 (11.2)		14 (8.1)							
	>1 year of clinical work experience, completed the first-year clinical competency training for nursing staff, and passed the review successfully	17 (17.3)		24 (13.9)							
	>2 years of clinical work experience, completed the second-year clinical competency training for nursing staff, and passed the review successfully	50 (51)		92 (53.2)							
	>3 years of clinical work experience, completed the third-year clinical competency training for nursing staff, and passed the nursing association's case report review successfully	12 (12.2)		26 (15)							
	>4 years of clinical work experience, completed the fourth-year clinical competency training for nursing staff, and passed the nursing association's administrative project review successfully	7 (7.1)		16 (9.2)							
**Emotional personality**								0.512 (1)			.47
	Positive emotions (optimistic character)	79 (80.6)		133 (76.9)							
	Negative emotions (pessimistic character)	19 (19.4)		40 (23.1)							

**Table 3:** Comparison of emotional exhaustion between the experimental group and control group.

Tests	Experimental group(n=98), mean (SD)	Control group(n=173), mean (SD)	*P* value
Workload	5.21 (1.11)	5.09 (1.13)	.51
Patients’ or relatives’ requirements	4.96 (1.19)	4.47 (1.30)	.39
Patient suffering	4.98 (1.11)	4.95 (1.25)	.24
Team collaboration problems	4.31 (1.09)	4.30 (1.12)	.80
Emotional dissonance	4.65 (1.38)	4.61 (1.30)	.77
Emotional exhaustion	4.80 (1.16)	4.78 (1.07)	.30

### Descriptive Statistics of Key Variables

In this study, the primary contributors to emotional exhaustion among nursing staff included heavy workloads, difficult patient interactions, and the distress of witnessing patient suffering. Specifically, “too many patients to care for” was scored the highest, at a mean of 5.41, in the workload category, indicating that the large number of patients significantly increased work stress, leading to emotional dissonance and eventual exhaustion. In the category of patient and family demands, “communicating with difficult or demanding patients” received a mean score of 5.13, highlighting the challenges nurses faced with handling difficult communication, which further exacerbates emotional exhaustion. Additionally, “witnessing patient pain and suffering” received a mean score of 5.30, reflecting the emotional toll on nurses who felt powerless to alleviate patient suffering, resulting in emotional buildup and exhaustion.

Regarding team cooperation, “lack of recognition for career development” received a mean score of 4.58, suggesting that the lack of acknowledgment for professional growth contributes to burnout and diminished enthusiasm for advancing in the nursing profession. In terms of emotional dissonance, “my emotions displayed for professional reasons are not consistent with my true feelings” received a mean score of 4.69, showing how the need to suppress personal emotions to meet professional standards leads to negative emotional buildup and exhaustion. Finally, “I feel exhausted at the end of the workday” received the highest mean score, at 5.30, in the emotional exhaustion category, underscoring the intense pressure and stress nurses experienced daily, leading to significant emotional exhaustion (Table 4).

**Table 4:** Questionnaire content related to each variable.

Variables		Questionnaire content	Results, mean (SD)^a^	Dimension score, mean^b^	
**Workload**					5.02
	w1	Insufficient time to accomplish tasks	4.59 (1.33)		
	w2	Too many patients	5.41 (1.27)		
	w3	Being assigned too many different tasks	5.25 (1.19)		
	w4	Lack of vacation time	4.87 (1.33)		
	w5	Having so much work that everything cannot be done well	4.98 (1.27)		
**Patients’ and relatives’ requirements**					4.81
	pr1	Dealing with difficult/demanding patients	5.13 (1.29)		
	pr2	Dealing with difficult/demanding relatives	5.05 (1.39)		
	pr3	Patients and/or family hostility or violence	4.59 (1.41)		
	pr4	Lack of cooperation with the patient	4.74 (1.37)		
	pr5	Conflict between the patient and their family	4.56 (1.40)		
**Patient suffering**					5.02
	ps1	Seeing the physical pain and suffering in patients	5.30 (1.24)		
	ps2	Physical deterioration in patients	5.22 (1.31)		
	ps3	No therapeutic hope	4.79 (1.33)		
	ps4	Worrying about patients’ loneliness	4.92 (1.24)		
	ps5	Being confronted with denial of the disease	4.89 (1.33)		
**Team collaboration problems**					4.35
	tcp1	Lack of recognition for work well done	4.53 (1.27)		
	tcp2	Experiencing conflicts with co-workers	4.14 (1.38)		
	tcp3	Unpleasant colleagues	4.18 (1.257)		
	tcp4	Disagreeing with physicians' practices	4.44 (1.26)		
	tcp5	Poor communication between coworker	4.21 (1.283)		
	tcp6	No reward for career development andadvancement	4.58 (1.384)		
**Emotional dissonance**					4.61
	ed1	The emotions that I feel in my job do not correspond to those Iwould like to feel.	4.57 (1.358)		
	ed2	My work situation brings me to experience emotions different than those I would like to feel.	4.61 (1.374)		
	ed3	I experience a discrepancy between the emotions I consider to beprofessional and what I feel.	4.57 (1.35)		
	ed4	The emotions I show in order to be professional are not consistent with my inner feelings.	4.69 (1.413)		
**Emotional exhaustion**					4.75
	ee1	I feel used up at the end of the workday.	5.3 (1.18)		
	ee2	I feel fatigued when I get up in the morning and have to face another day on the job.	5.28 (1.201)		
	ee3	I’ve become more callous toward people since I took this job.	4.63 (1.345)		
	ee4	Working with people directly puts too much stress on me.	4.53 (1.31)		
	ee5	I feel recipients blame me for some of their problems.	4.59 (1.332)		
	ee6	I feel frustrated by my job.	4.35 (1.267)		
	ee7	Working with people all day is really a strain for me.	4.59 (1.337)		

^a^Based on the Lister 7-point method (possible score ranging from 1 to 7).

^b^Mean of all item scores within a specific dimension (eg, stress, satisfaction, emotional state).

### Structural Equation Modeling and Partial Least Squares Results

This study used partial least squares to assess the reliability and validity of the collected questionnaire data. The analysis focused on internal consistency, convergent validity, and discriminant validity to ensure the accuracy of the data. Reliability was evaluated using Cronbach α and composite reliability. Both indicators demonstrated high reliability for all constructs, with Cronbach α values exceeding 0.86 and composite reliability values greater than 0.89, indicating strong internal consistency. Convergent validity was assessed using factor loadings and average variance extracted (AVE). Factor loadings primarily aim to explore whether the predefined factor model aligns with the collected observational data. This process examines whether the number of factors and the factor loadings of the observed variables meet expectations, thus validating the model’s consistency with the empirical data. All item factor loadings were greater than 0.7, showing a strong relationship between items and their respective constructs. The AVE values were greater than 0.5, confirming that the constructs captured sufficient variance from their items. Discriminant validity was evaluated by comparing the square root of the AVE values with the correlations between constructs. All constructs exhibited good discriminant validity, with the square root of AVE exceeding the correlations between constructs, indicating that these constructs are distinct from one another (Tables 5-7).

To enhance the statistical interpretability of the correlations between constructs, we calculated the *P* values for the correlation coefficients presented in Table 7 using Pearson correlation analysis. As shown in Table 7, given the sample size of 271, all interconstruct correlations reached statistical significance (*P*<.001).

**Table 5:** Questionnaire aspects for factor loading.

Questionnaire aspects (analysis code)		Factor loading
**Workload**		
	w1	0.860
	w2	0.896
	w3	0.874
	w4	0.925
**Patients’ and relatives’ requirements**		
	pr1	0.915
	pr2	0.913
	pr3	0.938
	pr4	0.924
	pr5	0.925
**Patient suffering**		
	ps1	0.904
	ps2	0.930
	ps3	0.901
	ps4	0.915
	ps5	0.909
**Team collaboration problems**		
	tcp1	0.917
	tcp2	0.906
	tcp3	0.759
	tcp4	0.931
	tcp5	0.836
	tcp6	0.882
**Emotional dissonance**		
	ed1	0.964
	ed2	0.969
	ed3	0.973
	ed4	0.937
**Emotional exhaustion**		
	ee1	0.785
	ee2	0.802
	ee3	0.891
	ee4	0.879
	ee5	0.884
	ee6	0.899
	ee7	0.880

**Table 6:** Reliability and validity of the questionnaire.

Questionnaire constructs	Cronbach α	CR^a^	AVE^b^
ed^c^	0.972	0.973	0.923
ee^d^	0.947	0.948	0.741
pr^e^	0.959	0.961	0.852
ps^f^	0.950	0.951	0.832
tcp^g^	0.934	0.936	0.761
w^h^	0.920	0.932	0.791

^a^CR: composite reliability.

^b^AVE: average variance extracted.

^c^ed: emotional dissonance.

^d^ee: emotional exhaustion.

^e^tcp: team collaboration problems.

^f^ps: patient suffering.

^g^pr: patients’ and relatives’ requirements.

^h^w: workload.

**Table 7:** Discriminant validity of the questionnaire, based on Pearson r correlation values (n=271).

Questionnaire constructs			ed^a^		ee^b^		pr^c^		ps^d^		tcp^e^		w^f^
**ed**													
	*r*	0.961		0.797		0.648		0.615		0.738		0.664	
	*P* value	—^g^		<.001		<.001		<.001		<.001		<.001	
**ee**													
	*r*	0.797		0.861		0.628		0.561		0.760		0.745	
	*P* value	<.001		—		<.001		<.001		<.001		<.001	
**pr**													
	*r*	0.648		0.628		0.923		0.604		0.670		0.686	
	*P* value	<.001		<.001		—		<.001		<.001		<.001	
**ps**													
	*r*	0.615		0.561		0.604		0.912		0.536		0.503	
	*P* value	<.001		<.001		<.001		—		<.001		<.001	
**Tcp**													
	*r*	0.738		0.760		0.670		0.536		0.872		0.617	
	*P* value	<.001		<.001		<.001		<.001		—		<.001	
**w**													
	*r*	0.664		0.745		0.686		0.503		0.617		0.889	
	*P* value	<.001		<.001		<.001		<.001		<.001		—	

^a^ed: emotional dissonance.

^b^ee: emotional exhaustion.

^c^tcp: team collaboration problems.

^d^ps: patient suffering.

^e^pr: patients’ and relatives’ requirements.

^f^w: workload.

^g^Not applicable.

## Discussion

### The Impact of Emotional Dissonance on Emotional Exhaustion

The findings support the hypothesis that emotional dissonance leads to emotional exhaustion among nursing staff, which is consistent with previous research showing that emotional dissonance depletes emotional resources and contributes to burnout.

### The Impact of Work-Related Factors on Emotional Dissonance

Workload had a significant positive effect on emotional dissonance in the control group (*t*_269_=2.257, *P*=.02) but not in the experimental group (*t*_269_=1.467, *P*=.14). Patient and family demands did not significantly affect emotional dissonance in either group. Caring for patients who were suffering showed a marginal effect in the experimental group (*t*_269_=1.948, *P*=.051) but was not significant in the control group. Teamwork issues significantly influenced emotional dissonance in both groups (*t*_269_=5.925, *P*≤.001)

### The Impact of Work-Related Factors on Emotional Exhaustion

Workload significantly impacted emotional exhaustion in both groups (experimental group: *t*_269_=4.283, *P*<.001; control group: *t*_269_=3.082, *P*=.002). The effects of patient and family demands and caring for suffering patients were not significant. Teamwork issues were significantly associated with emotional exhaustion in both groups (experimental: *t*_269_=2.239, *P*=.03; control: *t*_269_=2.522, *P*=.01).

### Moderating Effects of Listening to Podcasts on Work-Related Factors

None of the hypothesized moderating effects of podcast listening were statistically significant. The effects of workload, patient and family demands, caring for patients who are suffering, teamwork issues, and emotional dissonance on emotional exhaustion were not significantly moderated by podcast use (all *P* values >.05).

The model explained a substantial portion of the variance in emotional dissonance (experimental: R²=0.618; control: R²=0.629) and emotional exhaustion (experimental: R²=0.731; control: R²=0.711), but podcast listening did not significantly moderate the relationships among the studied variables (Table 8).

**Table 8:** Path coefficients.

Hypotheses	Experimental group			Hypothesis supported?	Control group				Hypothesis supported?
	Path coefficient	*t* (*df*)	*P* value		Path coefficient	*t* (*df*)	*P* value		
Emotional dissonance and emotional exhaustion	0.476	6.427 (269)	<.001	Yes	0.321	2.583 (269)	.01	Yes	
Patients’ and relatives’ requirement and emotional dissonance	0.123	1.135 (269)	.26	No	0.113	0.660 (269)	.51	No	
Patient suffering and emotional dissonance	0.173	1.948 (269)	.051	Yes	0.179	1.403 (269)	.16	No	
Team collaboration problems and emotional dissonance	0.507	5.925 (269)	<.001	Yes	0.348	2.961 (269)	.003	Yes	
Workload and emotional dissonance	0.125	1.467 (269)	.14	No	0.299	2.257 (269)	.02	Yes	
Patients’ and relatives/ requirements and emotional exhaustion	–0.004	0.044 (269)	.97	No	–0.021	0.167 (269)	.87	No	
Patient suffering and emotional exhaustion	0.045	0.853 (269)	.39	No	0.047	0.490 (269)	.32	No	
Team collaboration problems and emotional exhaustion	0.168	2.239 (269)	.03	Yes	0.293	2.522 (269)	.01	Yes	
Workload and emotional exhaustion	0.302	4.283 (269)	<.001	Yes	0.327	3.082 (269)	.002	Yes	

### Emotional Dissonance and Emotional Exhaustion

This study found a significant positive correlation between emotional dissonance and emotional exhaustion among nursing staff. Specifically, as emotional dissonance increases, so does the risk of emotional exhaustion. This pattern was observed in both the experimental group (nurses who regularly listened to podcasts) and control group (nurses who did not listen to podcasts). However, the emotional dissonance levels were slightly lower in the experimental group, suggesting that listening to podcasts may have a mild mitigating effect. The data showed that emotional dissonance is a significant predictor of emotional exhaustion. These findings are consistent with previous research, confirming that emotional dissonance depletes emotional resources, ultimately leading to emotional exhaustion among nursing staff.

### Workload, Emotional Dissonance, and Emotional Exhaustion

The results indicated significant positive correlations between workload and both emotional dissonance and emotional exhaustion. High workloads increased the risk of these outcomes, especially for nurses who did not listen to podcasts. In the experimental group, the path coefficient for the impact of workload on emotional dissonance was 0.125 (*t*_269_=1.467, *P*=.14), which was not significant, while in the control group, it was 0.299 (*t*_269_=2.257, *P*=.02), indicating a significant effect. Similarly, the path coefficients for the impact of workload on emotional exhaustion were 0.302 (*t*_269_=4.283, *P*<.001) in the experimental group and 0.327 (*t*_269_=3.082, *P*=.002) in the control group. These findings suggest that, although podcasts may help alleviate some stress, they are not sufficient to prevent emotional exhaustion under high workload conditions.

### Patient and Family Demands

The impact of patient and family demands on emotional dissonance and emotional exhaustion was minimal. Neither the experimental nor control group exhibited significant effects from these demands. For emotional dissonance, the path coefficient was 0.123 (*t*_269_=1.135, *P*=.26) in the experimental group and 0.113 (*t*_269_=0.660, *P*=.51) in the control group. For emotional exhaustion, the coefficients were –0.004 (*t*_269_=0.044, *P*=.97) in the experimental group and –0.021 (*t*_269_=0.167, *P*=.87) in the control group. This suggests that the professional skills and emotional management strategies of nursing staff effectively mitigate the impact of patient and family demands.

### Caring for Patients Who Are Suffering

In the experimental group, caring for patients who are suffering had an almost significant impact on emotional dissonance (path coefficient 0.173; *t*_269_=1.948, *P*=.051), but it was not significant in the control group (path coefficient 0.179; *t*_269_=1.403, *P*=.16). However, its impact on emotional exhaustion was not significant in either group. This suggests that, although caring for patients who are suffering may lead to emotional dissonance, it does not necessarily result in emotional exhaustion, possibly due to effective emotional management and professional support systems.

### Teamwork Issues

Teamwork issues showed a significant positive correlation with both emotional dissonance and emotional exhaustion. In the experimental group, the path coefficient for emotional dissonance was 0.507 (*t*_269_=5.925, *P*<.001), and for emotional exhaustion, it was 0.168 (*t*_269_=2.239, *P*=.03). In the control group, the coefficients were 0.348 (*t*_269_=2.961, *P*=.003) and 0.293 (*t*_269_=2.522, *P*=.01), respectively. These results indicate that issues such as poor communication and conflict within teams significantly increase stress, leading to emotional dissonance and exhaustion.

### Podcasts as a Moderating Factor

The study explored whether listening to podcasts could mitigate the effects of workload, patient demands, caring for patients who are suffering, and teamwork issues on emotional dissonance and exhaustion. However, the moderating effects of podcasts were generally not significant. For example, in the experimental group, the moderation of the impact of workload on emotional dissonance (*P*=.14) was not significant. Similarly, podcasts did not significantly reduce the impact of patient demands or teamwork issues on emotional exhaustion.

### Comparison With Previous Work

This study builds on existing research that has explored the relationship between emotional dissonance, workload, and emotional exhaustion among health care professionals. Previous studies, such as those by Bakker and Heuven [10] and Diestel and Schmidt [11], established a significant link between emotional dissonance and emotional exhaustion, highlighting the harmful effects of emotional labor on health care workers. Our findings confirm these studies, showing that emotional dissonance is a key predictor of emotional exhaustion in nursing staff. However, our research adds a new dimension by examining the potential moderating role of podcast listening in this relationship. Although the impact of emotional dissonance on exhaustion remains substantial regardless of podcast use, the slight alleviation observed in the experimental group suggests that podcasts may offer some emotional relief, an aspect not explored in earlier studies.

In terms of workload, our findings align with the existing literature, emphasizing the stress induced by high workloads in health care professionals. Studies by Glasberg et al [12] and Garrosa et al [13] highlighted that heavy workloads bring immense stress, leading to emotional exhaustion. Our study confirms these findings but also introduces the novel consideration of podcasts as a potential buffer. However, the effects of podcasts were insufficient and did not counteract the overwhelming impact of high workloads, suggesting the need for more comprehensive interventions.

Furthermore, unlike previous studies that mainly focused on the direct effects of patient demands and teamwork issues on emotional health, our study investigated these factors in the context of podcast use. Although the impact of patient demands on emotional dissonance and exhaustion was minimal, teamwork issues were found to have a significant impact, consistent with prior research. However, the moderating role of podcasts in mitigating these effects was limited, offering a nuanced understanding of the boundaries of digital media interventions in high-stress work environments.

### Factors Influencing the Study Results

The findings of this study indicate that the number of children the nurses had significantly differed between the 2 groups (*P*=.007), which may influence the interpretation of work stress and emotional exhaustion outcomes. Previous research has suggested that nurses with children may have heavier family responsibilities, leading to differences in their stress adaptation and emotional regulation compared with those without children. Particularly during the COVID-19 pandemic, nurses not only faced clinical stress but also the combined burden of childcare, household responsibilities, and health care duties, which could further contribute to increased emotional exhaustion.

However, we did not conduct a subgroup analysis to explore the impact of the number of children on the moderating effect of podcast listening. Therefore, future research should consider incorporating this variable into the model and using multivariate regression analysis or moderation effect analysis to control for potential confounding factors, ensuring the accuracy of study results.

### Strengths and Limitations

#### Strengths

This study has several strengths, including innovative use of podcasts, comprehensive analyses, and the ability to apply the findings in a practical setting.

This study is among the first to explore the use of podcasts as a tool for emotional support and stress management among nursing staff. By incorporating emerging digital media tools, the research offers new insights into how health care professionals can leverage technology to improve their emotional well-being. Regarding the analysis, the study thoroughly examines multiple sources of stress, including emotional dissonance, workload, patient demands, and teamwork issues, providing a holistic view of the factors leading to emotional exhaustion in nursing. The findings also have direct implications for health care institutions and policymakers, suggesting practical measures such as integrating podcast programs into emotional support strategies for nursing staff. Nevertheless, the generalizability of these findings to a broader nursing population should be interpreted with caution and warrants further validation in diverse clinical settings.

#### Limitations

At the same time, the study had some limitations, including sample representativeness, its cross-sectional design, the use of self-reported data, variations in podcast content and listening patterns by the participants, a potential lack of generalizability due to the cultural and linguistic context, and differences in the number of children the nurses had between the groups.

The study’s sample was limited to specific regions and health care institutions, which may affect the sample representativeness and restrict the generalizability of the findings. Different regions or types of health care institutions may have varying stressors and coping mechanisms. Additionally, the accessibility and adoption of digital interventions such as podcasts may differ across health care settings, further impacting the applicability of the results.

This cross-sectional study was conducted at a single point in time, making it challenging to capture the dynamic nature of stress and emotional exhaustion over time. Therefore, the long-term impact of podcast use on emotional well-being could not be assessed. Future longitudinal studies are needed to examine whether continued engagement with podcasts can lead to sustained emotional support and stress reduction among health care professionals.

The reliance on self-reported data introduces the possibility of subjective biases, such as social desirability or recall bias, which may affect the accuracy of the results. Participants may have provided responses based on perceived expectations rather than their actual experiences. Additionally, objective measures, such as physiological indicators of stress (eg, cortisol levels, heart rate variability), were not included, limiting the study’s ability to validate findings through biometric data.

The study did not account for the variation in podcast content, format, duration, and listening frequency, all of which could influence the results. Different types of podcasts may have varying effects on emotional support and stress reduction. For instance, content focused on relaxation techniques or peer support may provide stronger psychological benefits than general entertainment podcasts. Furthermore, the study did not assess individual differences in podcast engagement, such as the extent to which participants actively absorbed content versus using it as background noise. Future research should consider categorizing podcast types and evaluating their differential effects on emotional well-being.

The study was conducted in Taiwan, and cultural factors may influence how caregivers perceive stress and use podcasts. In collectivist cultures like Taiwan, social and familial expectations may shape nurses’ stress coping strategies differently than in individualistic cultures. Additionally, the availability of health-related podcasts in the local language may affect accessibility and engagement. The effectiveness of podcasts as an emotional support tool may vary depending on cultural attitudes toward digital health interventions and professional well-being. As a result, the findings may not be directly applicable to different cultural settings, and cross-cultural comparisons would be beneficial in future studies.

One of the limitations of this study was the significant difference in the number of children that nurses had between the groups, which may affect the internal validity of the research findings. Since nurses with children may experience higher levels of stress and emotional exhaustion, this factor should be considered when interpreting the results to account for its potential impact on the study findings. Future research may consider conducting subgroup analyses to explore the effects of varying family responsibilities on the moderating effect of podcast listening.

Additionally, the cross-sectional design limits the ability to infer causality. Future studies are recommended to adopt a longitudinal approach to further examine the long-term impact of podcast listening on emotional exhaustion.

### Future Research Directions

Therefore, future research should consider longitudinal study designs, expanding the sample diversity, analyzing podcast content, conducting comparative studies across health care professions, integrating podcasts with other digital tools, and conducting cross-cultural comparisons.

Longitudinal designs with sufficient time duration could track the impact of podcast use on emotional well-being over time. Furthermore, future longitudinal studies could help better understand the long-term impact of the variables examined in our research. This approach would help determine the long-term benefits or drawbacks of integrating podcasts into emotional support programs for health care professionals.

To enhance the generalizability of the findings, future studies should include more diverse samples, covering different regions, health care environments, and cultural contexts. This would provide a broader understanding of how podcasts can be utilized across various settings.

Future research should also analyze the specific content and quality of the podcasts used by health care professionals. Understanding which types of content are most effective in reducing stress and emotional exhaustion could lead to more targeted and effective podcast programs.

Exploring the use of podcasts across different health care professionals (eg, doctors, pharmacists, physical therapists) could reveal how podcast interventions can be customized to address the unique stressors faced by each group.

Combining podcasts with other digital tools (eg, mobile health apps or online counseling platforms) could create a more comprehensive emotional support system for health care workers. Future research should explore the synergies of using multiple digital resources for stress management.

Comparative studies between different countries and cultures would help determine how cultural factors influence the acceptance and effectiveness of podcasts as a stress management tool. This could lead to more culturally sensitive interventions in global health care settings.

### Conclusion

This study highlights the significant impact of work-related stress on emotional exhaustion among nursing staff and explores the potential of podcasts as a digital intervention to mitigate these effects. The findings indicate a strong association between higher work stress and increased emotional exhaustion, which poses critical challenges for both the well-being of nursing professionals and the quality of patient care. However, the findings of this study also suggests that regular engagement with podcasts, particularly those focused on emotional management and stress relief, may provide a beneficial coping mechanism, contributing to reduced emotional exhaustion among nurses.

The results provide evidence supporting the integration of podcasts as a supplementary psychological support tool within health care institutions. By incorporating digital mental health interventions such as podcasts, institutions can offer accessible and flexible psychological support, ultimately improving job satisfaction, emotional resilience, and overall well-being among nursing staff. Additionally, the study underscores the need for policy initiatives to actively promote and support digital interventions aimed at improving the mental health and professional development of health care workers.

Despite these insights, certain limitations must be acknowledged. The study identified a significant difference in the number of children between groups, which may influence stress coping mechanisms and the moderating effect of podcast listening. Future research should control for this variable to ensure an accurate evaluation of podcast interventions. Moreover, although podcasts may serve as a viable supplementary intervention, their moderating effect remains limited, indicating the need for further exploration into comprehensive psychological support strategies.

Future studies should investigate the long-term effects of podcast engagement, assess its efficacy across diverse health care settings and cultural contexts, and explore potential synergies with other digital or traditional psychological support programs. The integration of podcasts and similar digital tools represents a promising avenue for enhancing the psychological support systems available to nursing professionals, potentially improving health care outcomes and bolstering the resilience of the health care workforce.

## Abbreviations

AVE: average variance extracted
